# Nanogap structures for molecular nanoelectronics

**DOI:** 10.1186/1556-276X-7-113

**Published:** 2012-02-09

**Authors:** Paolo Motto, Alice Dimonte, Ismael Rattalino, Danilo Demarchi, Gianluca Piccinini, Pierluigi Civera

**Affiliations:** 1 Department of Electronics, Corso Duca degli Abruzzi 24, 10129 Turin, Italy; 2 Italian Institute of Technology, IIT@Polito Center, Corso Trento 21, Turin, Italy

## Abstract

This study is focused on the realization of nanodevices for nano and molecular electronics, based on molecular interactions in a metal-molecule-metal (M-M-M) structure. In an M-M-M system, the electronic function is a property of the structure and can be characterized through I/V measurements. The contact between the metals and the molecule was obtained by gold nanogaps (with a dimension of less than 10 nm), produced with the electromigration technique. The nanogap fabrication was controlled by a custom hardware and the related software system. The studies were carried out through experiments and simulations of organic molecules, in particular oligothiophenes.

## 1 Introduction

Electrical nanogap devices are emerging because of their possibility to be the building blocks for connecting [1], analyzing [2], and using molecules, and so for implementing nano-metric electronic devices [3]. The main advantage of these systems is, in general, the ability to measure and to transduce events of specific molecules into useful electrical signals [4]. As a consequence, nanogaps have nowadays a high level of interest in research. There are a lot of techniques for obtaining nanogaps, but a process to totally control the gap size has not been found yet. Electromigration effect is the simplest technique useful for obtaining the break of the two terminals structures where the nanogap is built [5,6]. Electro-induced break junction (EIBJ) can generate an instantaneous and random break, but to obtain reproducible and stable devices it is very important to control the width of the nanogap [7,8]. For this reason, the quantity of current used to stimulate the electromigration effect must be controlled with a custom feedback circuit that manages all the fabrication steps. The authors defined a method for producing nanogaps inside gold structures, and the controlled use of the electromigration enabled to build gaps under ten nanometers.

## 2 Experimental section

### 2.1 Realization of the chip

The electromigration is mainly dominated by the current density [9] and by the temperature of the wire [10]. Both these quantities can be controlled by a proper geometry of the probe [11] and by the applied voltage waveform [12]. Using electromigration as technique for creating nanogaps, the wire has to have optimized geometries to facilitate the phenomenon and make it more controllable. From this point, to avoid a too high input current, it is necessary to have a small section of the wire [11]; moreover, if the section of the wire is too small, the thermal conductance decreases and the temperature of the wire tends to become excessive, leading to the melting of the wire.

**Figure 1:**
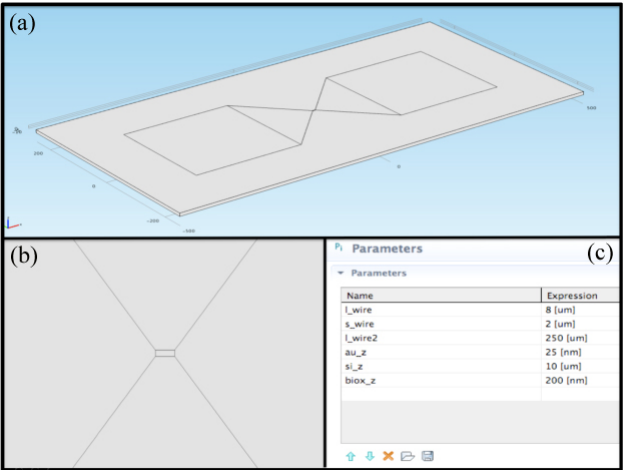
**Comsol model of the probe**. **(a) **Geometry of the probe; **(b) **single wire level; **(c) **best parameters for generating a sharp temperature profile: wire length l_wire = 8 *μm*; wire width s_wire = 2 *μm*; wire thickness au_z = 25 nm; substrate thickness bios_z = 200 nm.

For these reasons, it is fundamental to have a model that can simulate the behavior of the physical phenomena during the electromigration. In particular, it is interesting to know and to anticipate the temperature inside the gold wire and for this it is necessary to model the geometry of the probe with a software simulation tool. The objective is therefore to achieve a wire section as small as possible, to trigger the electromigration also at lower current, obtaining a better control and a more regular shape. Figure 1a shows the geometry of our probe described in Comsol Multi-physics, while Figure 1b shows the single wire. To evaluate the temperature behavior during the voltage application, we performed a large number of simulations using different values of wire length, keeping in mind that there is a lower limit for the length of the wire that will allow electromigration to occur (the Blech length [13-15]). Figure 1c shows the best parameters for generating a temperature profile quite sharp in a way that should be possible to focus the electromigration phenomenon in the center of the wire. The temperature profile along wire length was modeled with the equations:

(1) $$Q=\rho C_{p}\frac{\partial T}{\partial t}-\nabla \left(K\nabla T\right)$$

(2) $$Q=\sigma \text{|}\nabla V{\text{|}}^{\text{2}}$$

(3) $$\vec{J}=\sigma \nabla V$$

**Figure 2:**
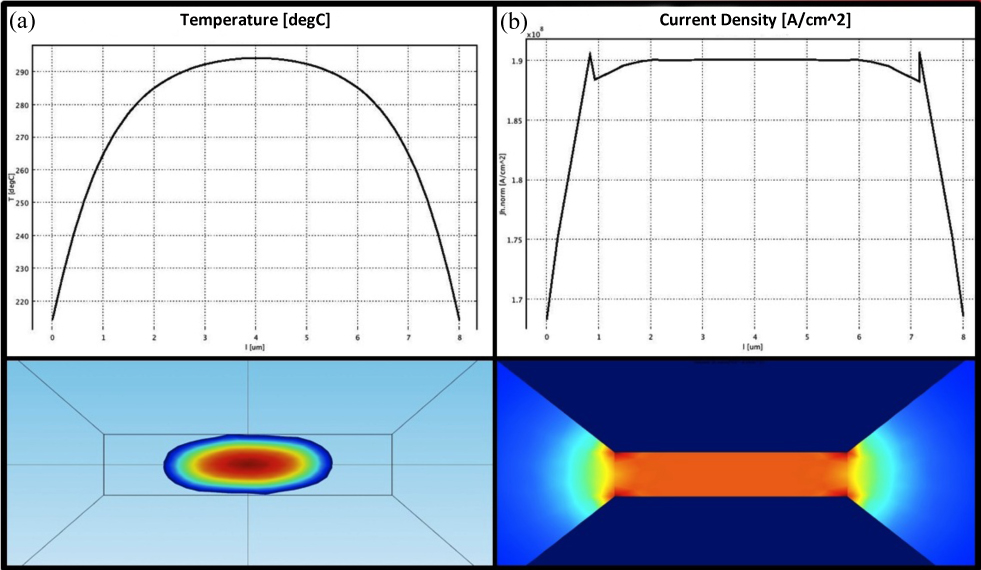
**Results of the electrothermal simulation made with Comsol**. **(a) **Temperature profile; **(b) **current density profile.

Equation (1) is the law of conservation of energy: *Q *is the power transferred to a point, *ρ *is the mass volume density, *C_p _*is the heat capacity, *T *is the temperature, *t *is the time and *K *is the thermal conductivity. Equation (2) gives the power dissipation (Joule effect): *σ *is the electrical conductivity and *V *is the electrical potential. Finally, Equation (3) is the Ohm's law in local form: *J *is the current density. All the equations are related to an infinitesimal point of space. Comsol uses the nodes of the mesh geometry to make a spatial sampling and integrates the equations in the volume using the nodes as points of integration: if the sampling is quite dense, the error is negligible. As result we have obtained the plot shown in Figure 2a that represents the temperature variation in the wire length. The geometry created also allows a uniform distribution of current density (Figure 2b).

**Figure 3:**
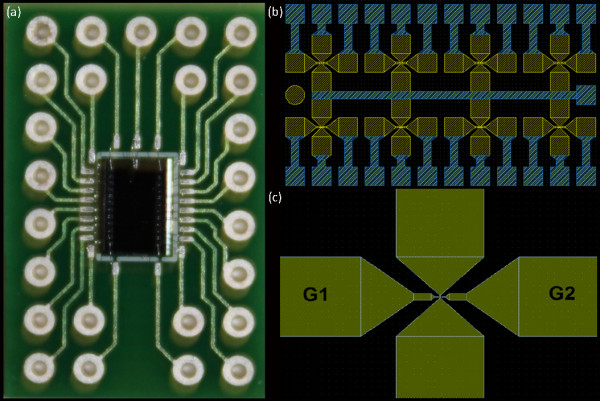
**The chip. Picture of the final chip containing the eight probes**. **(a) **The chip bonded on the PCB; **(b) **layout of the chip; **(c) **zoom of one of the probes: G1 and G2 indicate the pads of the gates.

For having a useful platform where to produce the nanogaps, a silicon chip was realized, containing eight gold probes as shown in Figure 3b, each of these connectable by bonding. In this way, it is possible to realize on the same chip eight nanogap structures, and each one is independent, so an high number of measurements is individually achievable. The final dimension of the chip is 2.4 × 4.1 mm, giving the possibility to insert it in heads of instruments as a cryostat or FESEM/AFM/STM microscopes, for doing for example measurement in vacuum and at very low temperatures. The chip is also ready to be wire bonded to a PCB (Figure 3a). It is possible to perform wet analysis too, for molecule characterizations, just spinning on the chip the solution that has to be measured. Obviously molecules in solutions must have some suitable sites for bonding with gold, such as thiol groups, in this way it is possible to obtain the desired M-M-M structure.

**Figure 4:**
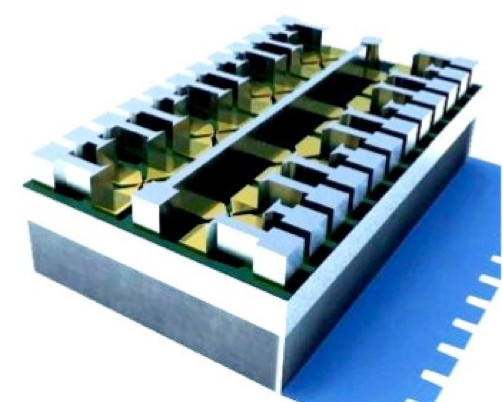
**Chip structure representation**. 3D representation of the final chip with the gold pads.

The realization of the chip starts from a silicon wafer capped by 200 nm of *SiO*_2_; the wafer is, then, inserted in a plasma oxygen machine for increasing the oxygen atoms concentration on the surface. Hydroxylation process is developed with a piranha solution, and so, after rinsing and drying, the surface of the wafer exposes -OH groups, fundamental for the anchoring of the organic compound that is evaporated on. In fact, to promote the adhesion of gold on the *SiO*_2 _surface, an insulating layer of MPTMS

(3-Mercaptopropyl)trimethoxysilane is then deposited [16,17]. The insulation property is important because has to be avoided any alternative path of current, that has to flow through the gold layer only. After, a gold film of 25 nm is grown on the MPTMS layer using an EVA600 evaporator. The wafer is inserted in a EVG-SUSS MA6 where a photolithographic mask process is performed employing a positive photoresist, afterwards the gold etching is done thanks to Iodine/Potassium solution. A second photolithographic process is performed through a second mask that allows the realization of the chip's pads, built with a thin layer of titanium of 100 nm and an aluminum layer of 700 nm (see Figure 4).

### The custom hardware

**Figure 5:**
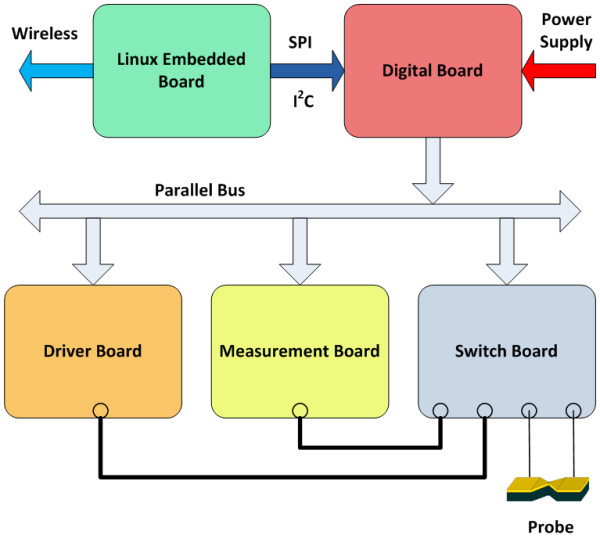
**Block diagram of the electronic system**. The system and its modularity.

To control the experiments, a custom electronic board, connected to a Linux-embedded system, has been realized. To provide the current density of 10^8^*A*/*cm*^2^, needed for the activation of the electromigration process, the circuit must be able to supply a current of at least 50 mA, because of the dimensions of realized geometries. The front-end must also be able to measure the real time current flowing in the wire, to evaluate resistance variations, from hundreds of mA (when the current is high and the break is not yet created) to some pA (for measuring the tunnel current inside the nanogap). The block diagram of the system is showed in Figure 5, where it is possible to see that the gold probe is connected to the circuit that receives the signal from the embedded Linux (analog input) and generates the desired current for inducing the electromigration. The current in the probe, and so the resistance, is measured. These values are sent to the embedded Linux in which are implemented the algorithms for controlling the current to inject into the probe. In this way, the fabrication of the nanogap is checked, avoiding thermal runaway that can create too large breaks [18-22], not useful for the desired applications.

The custom system consists of a *driver board *that hosts a digital-to-analog converter that realizes the input voltage waveform; a *measurement board *composed by a transimpedance amplifier with variable feedback, in order to measure a wide range of currents, and an analog-to-digital converter; a *switch board *to allow the connection of external instrumentation; a *digital board *that provides electrical power supplies and the bus connection between all boards. The embedded Linux system is built with a real-time custom kernel, so that the electronic components of the other boards are driven in a deterministic way. We have developed a wireless connection between this board and a host computer for sending the experimental data. This solution allows also the use of the system in chambers where a wire interconnection can create difficulties.

### The custom algorithm

**Figure 6:**
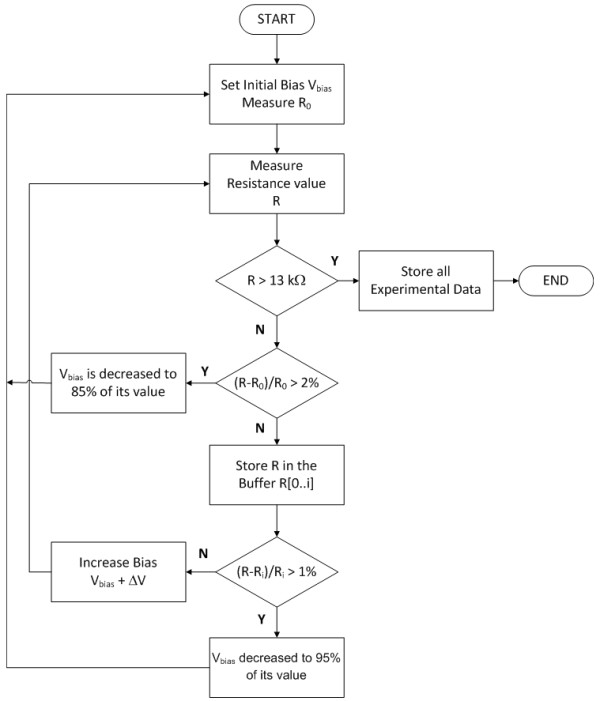
**Flowchart of the algorithm governing the feedback-controlled electromigration process**. The two feedback mechanisms: the first one is used to avoid the thermal runaway and the second one is used to control the electromigration effect.

**Figure 7:**
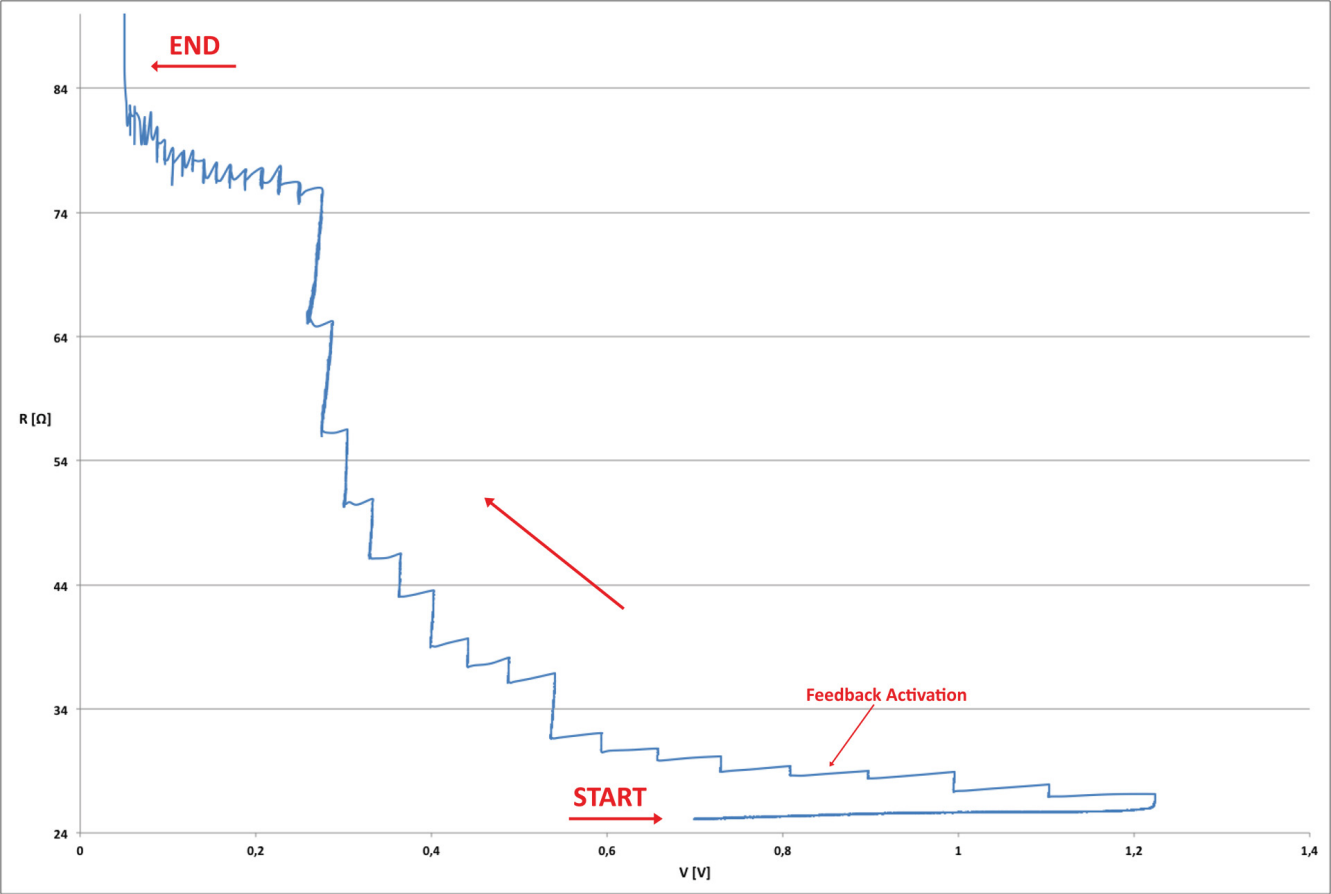
**Monitoring plot of the probe resistance**. R/V plot of an EIBJ experiment.

Nanogaps are produced using a specific algorithm, customized for an optimal nanogap fabrication and with several goals: current management, feedback controlled breaking, temperature control for avoiding the thermal runaway. A simplified schematic of the process flow is reported in Figure 6. The software controls the voltage *V*_bias _applied to the probe and stops when the resistance exceeds the value of 13 *k*Ω, that means that the nanogap is produced. In fact this resistance value is about the inverse of the quantum conductance 2*e*^2^/*h *= 77.6 *μS *and represents the conductance of a single atom of gold placed between two electrodes [23]. As it is possible to see always in Figure 6, there are two feedback mechanisms. The first one performs an absolute control over the initial resistance *R*_0_, and when (*R *- *R*_0_)/*R*_0 _*>*0.02 the *V*_bias _is set to the 85% of its value; this is done for controlling the temperature of the wire and for preventing melting and surface tension effects, that can be the cause of much larger gaps and gold island formation [18-20]. The second mechanism performs a check of the resistance value relative to a circular buffer of n samples, when (*R *- *R_i_*)/*R_i _>*0.01, with *R_i _*the average of the last *i *samples, the *V*_bias _is decreased to the 95% of its value. The second feedback mechanism performs a check of the resistance in order to control the electromigration effect: the increase of the resistance is no longer linear as in the Joule Heating, but exponential, and we need to promptly react to stop the phenomenon. We also noted in many experiments performed by us, that, after the activation of the feedback, the resistance value decreases due to the acceleration of the grain growth by Joule heating of the wire [24]. In this case, the first mechanism becomes useless and then the process is controlled only by the second one. Figure 7 shows the probe resistance as a function of the voltage applied: when the temperature increases, the resistance of the wire tends to increase too.

The higher resistance causes a current reduction, but increasing the voltage in this case creates a mechanism by which the current flow tends to be constant. In fact the first increase in resistance (linear growth) is only due to this heat effect, but, when the temperature reaches high values and the current density is near to 10^8 ^A/cm^2^, the electromigration starts (exponential growth) and the structure begins to change.

**Figure 8:**
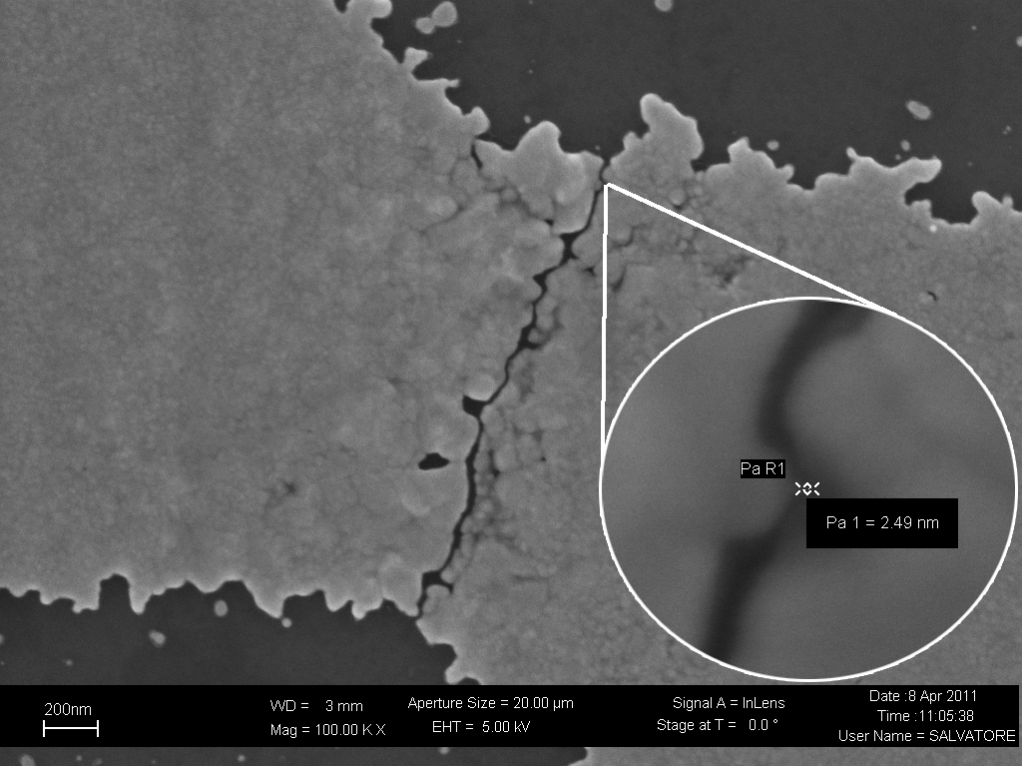
**FESEM image of a nanogap**. 2.49 nm nanogap. Image captured by Field Effect Scanning Electron Microscope (FESEM), 100 k*× *magnification (700 k× magnification for the zoom zone).

The experimental time of electromigration has been estimated to be about 50-60 min. All the experimental data obtained by the electromigration tests are stored in an internal database in the Linux board, but on the host computer too, that collects information through the wireless connection. This makes possible to generate very accurate statistics. To evaluate the outcome of our experiments, we performed the analysis of gap widths with a FESEM microscope. Example of a fabricated nanogap is shown in Figure 8.

## 3 Results

**Figure 9:**
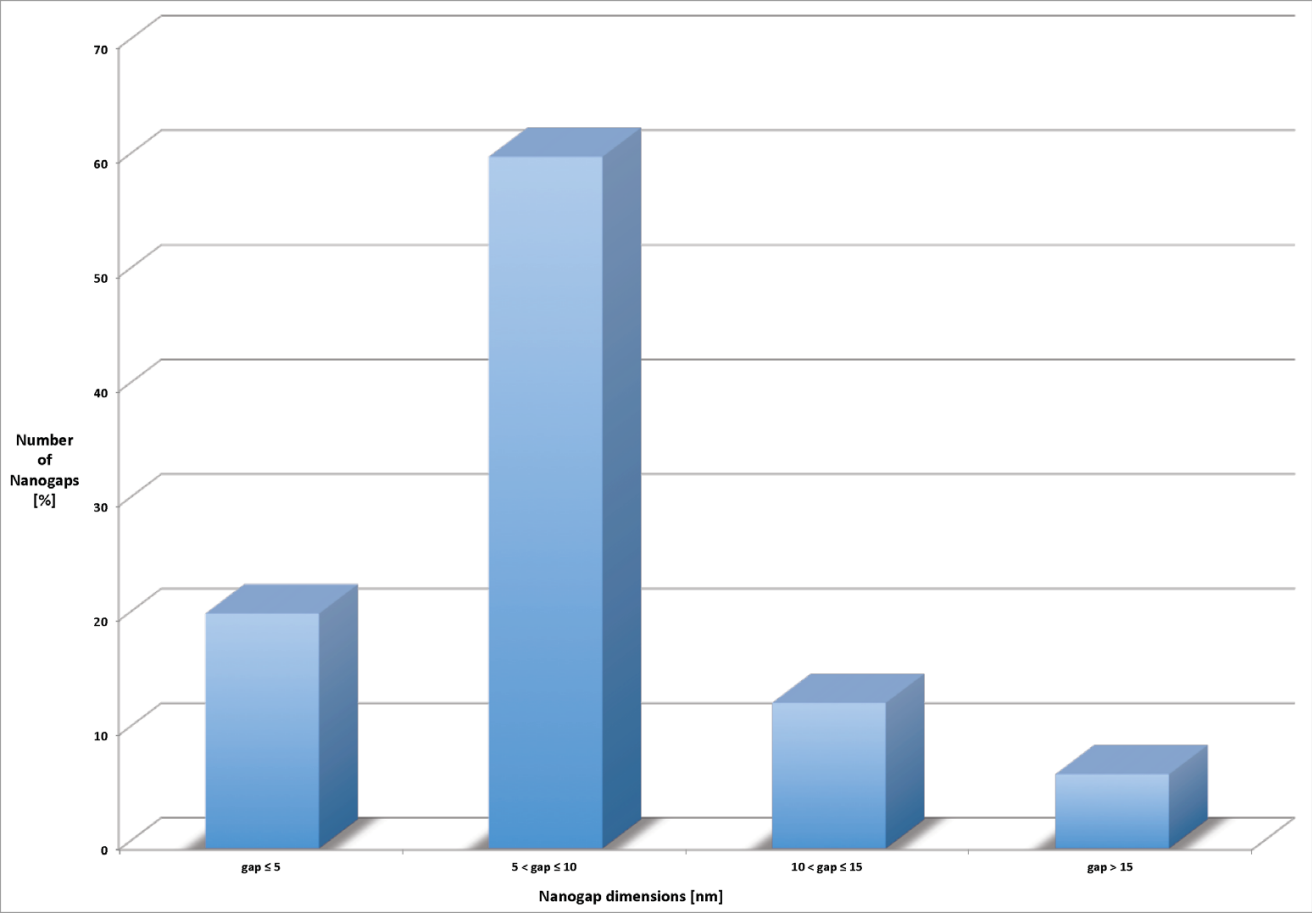
**Plot of the distribution of the nanogap dimension**. Statistical dispersion of nanogap dimensions, 80% of them are under 10 nm.

The method used to fabricate nanogaps, through these "ad hoc" software and electronic circuit, has produced nanogaps under 3 nm as the one shown in Figure 8. It is interesting to observe that the gap has an almost constant width for a relative long path. Analyzing the experimental results, fabricated nanogaps show an average dimension of less than 10 nm. Statistical analysis about the final dimension of the gaps confirm that the authors are now able to create nanogaps under 10 nm with an high reproducibility (Figure 9), in fact about the 80% of the nanogaps are under 10 nm.

**Figure 10:**
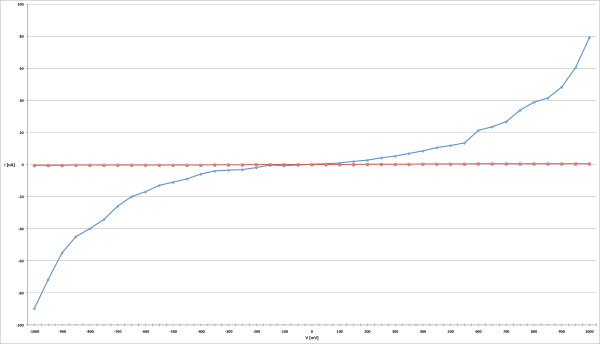
**I/V characteristic of the M-M-M system**. I/V plot of the M-M-M system with the oligothiophene molecule inside the gap (blue line) and the THF solvent (red line). The concentration of the solution contain the molecules deposited on the chip was 100 nM. The size of the nanogap is 4,7 nm.

To evaluate the use of nanogaps as electrodes for molecular electronics, a solution of oligothiophenes molecules (2,2':5',2":5",2"'-bis-quaterthiophene) in TetraHydroFuran (THF) has been deposited using the method of spin coating. The choice of this type of molecules is due to the presence of a sulfur atom in the aromatic ring: it binds easily to the gold of the nanogap electrodes. After the insertion in the nanogaps, the molecular solution was characterized by current-voltage (I/V) measurements (Figure 10), made by a Sub-Femtoamp Remote SourceMeter (Keithley 6430). The blue line, reported in Figure 10, shows a profile with the expected trend (see the characterization of molecular wires in [25-29]). The red line represents the characteristic of the solvent THF in an open nanogap: the values reported show that the contribution of both the solvent and the tunneling current dependent on the size of the nanogap are negligible. It is interesting to mention that the curve is asymmetric, indicating that the contacts between the molecule and the electrodes are not perfectly symmetric [30-32].

The molecular length plays a key role in the electrical conduction.

**Figure 11:**
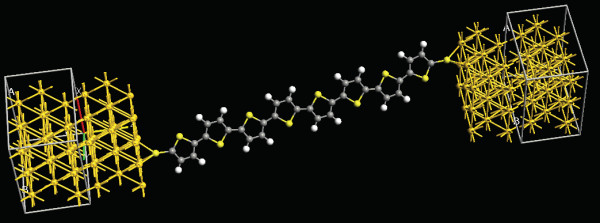
**Orientation of the oligothiophene molecule**. *Ab initio *simulation of the molecule placed between the electrodes. The figure shows the orientation of the molecule after the optimization.

Experiments have found that the conductance *G *decreases exponentially with molecular length *L *[33] and can be described by

(4) $$G=Ae^{-\beta L}$$

where *A *is a constant and *β *is a decay constant varying between 0.09 and 0.16 *Å*^-1 ^for the oligothiophenes [28,29]. Figure 11 shows an *ab initio *simulation of the molecule placed between the electrodes where the molecule orientation was performed with the software Gaussian 09. Different orientations are possible, but less probable. Moreover, our simulations show a variation of the current less than 30% respect to the optimized case, keeping the same shape of the I/V curve.

## 4 Conclusions

A system composed by a software interface and an electronic control circuit for the nanogap realization has been implemented, and all the technological steps for arriving at the final nanogap production has been presented in this study. The probe geometries were optimized through electrothermal simulations performed with the COMSOL Multi-physics software. The method applied demonstrated the possibility to build nanogaps under 3 nm with controlled feedback, having a good statistical yield with about the 80% of the nanogaps below 10 nm (Figure 9). In the experimental phase an oligothiophene molecule was successfully inserted in the nanogap, producing a first Metal-Molecule-Metal system, and it was characterized by current-voltage (Figure 10) measurements, taking into account that the coupling between metal and molecule plays a key role. Future study will be focused on the optimization of the system for the realization of integrated molecular devices.

## Competing interests

The authors declare that they have no competing interests.

## Authors' contributions

PM made the electronic system, the EIBJ experiments, developed the RT kernel and integrated the algorithm into the software. AD realized the chip in clean room. IR performed the electrothermal simulation. DD, GP and PC designed the experimental idea and gave technical background and support concerning the field of study. All authors read and approved the final manuscript.
